# Geographical tracking and mapping of coronavirus disease COVID-19/severe acute respiratory syndrome coronavirus 2 (SARS-CoV-2) epidemic and associated events around the world: how 21st century GIS technologies are supporting the global fight against outbreaks and epidemics

**DOI:** 10.1186/s12942-020-00202-8

**Published:** 2020-03-11

**Authors:** Maged N. Kamel Boulos, Estella M. Geraghty

**Affiliations:** 1 School of Information Management, Sun Yat-sen University, East Campus, Guangzhou, 510006 Guangdong China; 2 Esri (Environmental Systems Research Institute), 380 New York St, Redlands, CA 92373 USA

**Keywords:** COVID-19, SARS-CoV-2, GIS

## Abstract

In December 2019, a new virus (initially called ‘Novel Coronavirus 2019-nCoV’ and later renamed to SARS-CoV-2) causing severe acute respiratory syndrome (coronavirus disease COVID-19) emerged in Wuhan, Hubei Province, China, and rapidly spread to other parts of China and other countries around the world, despite China’s massive efforts to contain the disease within Hubei. As with the original SARS-CoV epidemic of 2002/2003 and with seasonal influenza, geographic information systems and methods, including, among other application possibilities, online real-or near-real-time mapping of disease cases and of social media reactions to disease spread, predictive risk mapping using population travel data, and tracing and mapping super-spreader trajectories and contacts across space and time, are proving indispensable for timely and effective epidemic monitoring and response. This paper offers pointers to, and describes, a range of practical online/mobile GIS and mapping dashboards and applications for tracking the 2019/2020 coronavirus epidemic and associated events as they unfold around the world. Some of these dashboards and applications are receiving data updates in near-real-time (at the time of writing), and one of them is meant for individual users (in China) to check if the app user has had any close contact with a person confirmed or suspected to have been infected with SARS-CoV-2 in the recent past. We also discuss additional ways GIS can support the fight against infectious disease outbreaks and epidemics.

## Introduction

In December 2019, a new virus (initially called ‘Novel Coronavirus 2019-nCoV’ and later renamed to SARS-CoV-2) causing severe acute respiratory syndrome (coronavirus disease COVID-19) emerged in Wuhan, Hubei Province, China [1], and rapidly spread to other parts of China and other countries around the world, despite China’s massive efforts to contain the disease within Hubei.

Compared to the 2002/2003 SARS-CoV and the 2012–2014 MERS-CoV (Middle East Respiratory Syndrome-related coronavirus), the COVID-19 coronavirus spread strikingly fast. While MERS took about two and a half years to infect 1000 people, and SARS took roughly 4 months, the novel SARS-CoV-2 reached that figure in just 48 days. On 30 January 2020, the World Health Organization (WHO) declared that the new SARS-CoV-2 coronavirus outbreak constitutes a Public Health Emergency of International Concern (PHEIC) [2].

As with the original SARS-CoV epidemic of 2002/2003 [3] and with seasonal influenza [4, 5], geographic information systems (GIS) and methods, including, among other application possibilities, online real- or near-real-time mapping of disease cases and of social media reactions to disease spread, predictive risk mapping using population travel data, and tracing and mapping super-spreader trajectories and contacts across space and time (see, as an example, the first diagram in [6]), are proving indispensable for our timely understanding of the new disease source, dynamics and epidemiology, and in shaping our effective response to it.

Indeed, health professionals have long considered conventional mapping, and more recently geographic information systems (GIS), as critical tools in tracking and combating contagion. The earliest map visualisation of the relationship between place and health was in 1694 on plague containment in Italy [7]. The value of maps as a communication tool blossomed over the next 225 years in the service of understanding and tracking infectious diseases, such as yellow fever, cholera and the 1918 influenza pandemic. From the 1960s, when computerised geographic information systems were born, the possibilities for analysing, visualising and detecting patterns of disease dramatically increased again. A 2014 review of the health GIS literature found that 248 out of 865 included papers (28.7%) focused on infectious disease mapping [8].

Since then we have seen a revolution in applied health geography through Web-based tools [9, 10]. Now, as we deploy these tools to protect human lives, we can ingest big data from their sources and display results in interactive and near-real-time dashboards. These online dashboards have become a pivotal source of information during the COVID-19 outbreak.

This paper offers pointers to, and describes, a range of practical online/mobile GIS and mapping dashboards and applications for tracking the coronavirus epidemic and associated events as they unfold around the world. Some of these dashboards and applications are receiving data updates in near-real-time (at the time of writing), and one of them is meant for individual users (in China) to check if the app user has had any close contact with a person confirmed or suspected to have been infected with SARS-CoV-2 in the recent past. We also briefly discuss additional ways GIS can support the fight against infectious disease outbreaks and epidemics.

## Johns Hopkins University Center for Systems Science and Engineering dashboard

**Fig. 1:**
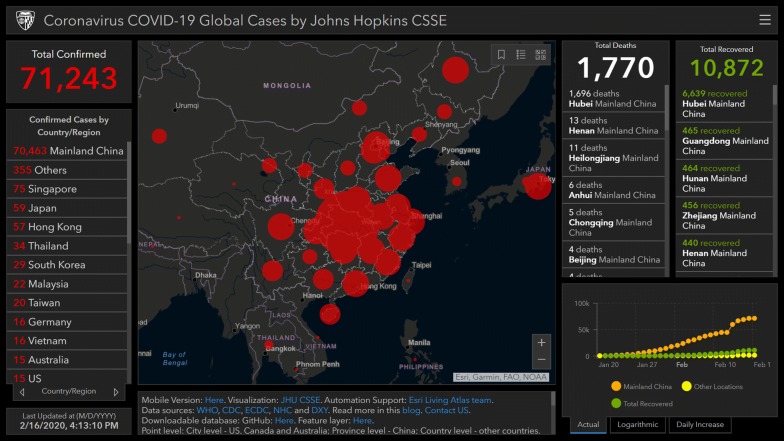
Johns Hopkins University CSSE is tracking the spread of SARS-CoV-2 in near real time with a map-centric dashboard (using ArcGIS Online) that pulls relevant data from the WHO, US CDC (Centers for Disease Control and Prevention), ECDC (European Centre for Disease Prevention and Control), Chinese Center for Disease Control and Prevention (CCDC), NHC (China’s National Health Commission), and Dingxiangyuan (DXY, China). Screenshot date: 16 February 2020

When disease can travel so quickly, information has to move even faster. This is where map-based dashboards become crucial [11]. At the time of this writing in mid-February 2020, seven coronavirus dashboards are among the top ten requested applications from Esri ArcGIS Online service, accumulating over 160 million views. First published on 22 January 2020 in response to escalating pandemic fears in late January 2020, the Johns Hopkins University’s Center for Systems Science and Engineering (JHU CSSE) dashboard leads the pack, garnering 140 million views. Developed by Lauren Gardner (an epidemiologist) and her team from the JHU CSSE, the dashboard went viral with hundreds of news articles and shares on social media (Fig. 1) [12]. This intense response to the JHU CSSE and other dashboards shows how eager people are to track health threats. Anyone with Internet access can learn, in a few short clicks, a tremendous amount of information about the COVID-19 virus from these resources.

The JHU CSSE dashboard’s interactive map locates and tallies confirmed infections, fatalities and recoveries. Graphs detail virus progress over time. Viewers can see the day and time of the most recent data update and data sources. The dashboard’s five authoritative data sources include World Health Organization (WHO), US Centers for Disease Control and Prevention, National Health Commission of the People’s Republic of China, European Centre for Disease Prevention and Control, and the Chinese online medical resource DXY.cn. The dashboard provides links to these sources and others. A blog post [13] details this work. The corresponding data repository is accessible as Google sheets in GitHub [14].

Web services allow GIS users to consume and display disparate data inputs without central hosting or processing to ease data sharing and speed information aggregation. In the first dashboard iteration, from January 22 through January 31, 2020, the Hopkins team manually updated data twice per day. In February 2020, Esri’s ArcGIS Living Atlas team assisted them in adopting a semi-automated living data stream strategy to update the dashboard. It primarily relies on the DXY.cn data resource, which updates every 15 min for case reports at provincial and country levels. However, Lauren Gardner’s blog [13] notes that for countries outside of China, other data resources were quicker to update than DXY.cn, so those case counts are manually updated throughout the day. Current feature layers are freely accessible in the ArcGIS Living Atlas [15].

Yet, the COVID-19 outbreak has been difficult to monitor. As Gardner explains, “*it is especially challenging to collect good data at a fine spatial resolution, which is what most people want to know, and without having travel data in real time that captures these altered mobility patterns, it is hard to assess what the geographic risk profile will look like moving forward*” [16].

The JHU CSSE dashboard (at the time of writing) lacks archiving services for full retrospective map visualisation of data from previous days. As far as the latter are concerned, the dashboard only offers timeline charts of total confirmed cases (grouped into ‘mainland China’ and ‘other locations’) and total recovered cases. But one is unable to retrieve and display detailed map snapshots in time (by individual country/region and Chinese province), e.g., to see how the coronavirus world distribution map looked in the past on a specific day, such as 25 January 2020. The dashboard developers are encouraged to compile and make such interactive daily map snapshots permanently accessible online for future reference after the epidemic has gone, as a service to public health researchers and professionals worldwide.

## The World Health Organization dashboard

**Fig. 2:**
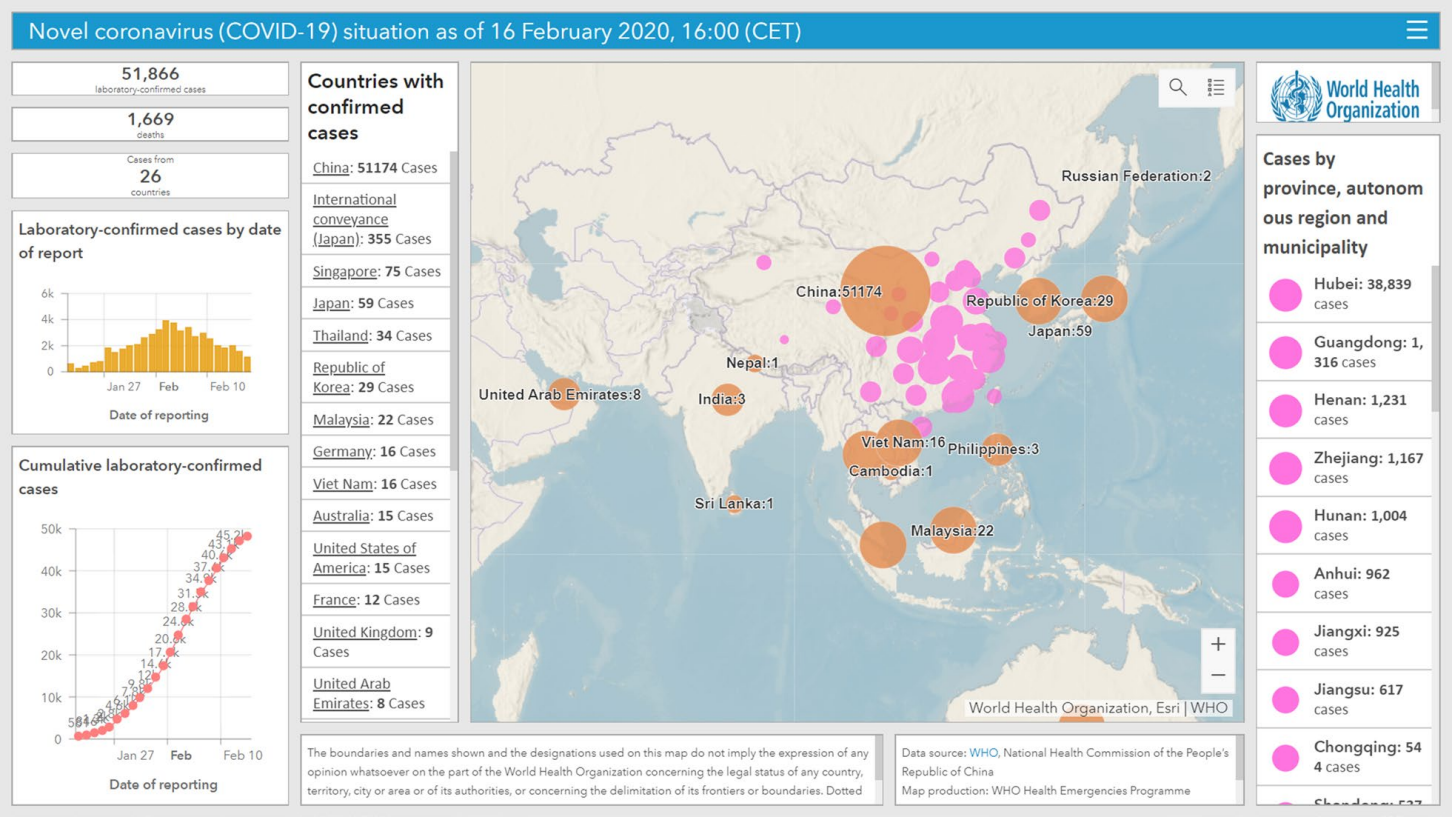
The WHO COVID-19 situation dashboard. Screenshot date: 16 February 2020

The WHO directs and coordinates international health, combating communicable diseases through surveillance, preparedness and response, and applying GIS technology to this work. On 26 January 2020, the WHO unveiled its ArcGIS Operations Dashboard for COVID-19, which also maps and lists coronavirus cases and total number of deaths by country and Chinese province, with informational panels about the map and its data resources (Fig. 2) [17].

Prior to 18 February 2020, the WHO and JHU CSSE dashboards had some interesting differences. Each had a vastly different total case count as can be seen in Figs. 1 and 2 (both taken on 16 February 2020). The WHO dashboard reflected laboratory-confirmed cases, whereas JHU CSSE included cases diagnosed using a symptom array plus chest imaging (accounting for some 18,000 additional reports). However, as of 19 February 2020, both dashboards are in sync, displaying similar total case counts.

The WHO dashboard includes an epidemic curve up front, showing cases by date of reporting. Putting the epi curve visualisation above the cumulative cases graph provides important information about outbreak progression, and may decrease fear since we can see a steady decline in the total number of new cases per day since 4 February 2020 (except for a spike on 13 February 2020, when more than 15 K cases were added after China started to include ‘clinically diagnosed’ cases and not just laboratory-confirmed cases in its figures).

A ‘hamburger’ menu at the top right corner of the WHO dashboard provides links to additional information about COVID-19 and leads to an interactive Web map that puts COVID-19 into context among other WHO-monitored health emergencies, such as dengue fever, Rift Valley fever and West Nile fever [18].

The WHO is updating its COVID-19 dashboard automatically using ArcGIS GeoEvent Server to push updates to a single feature service multiple times per day. The WHO dashboard optimisation measures include moving tiled data off its server and into ArcGIS Online tiled services to benefit from Esri’s content delivery network. This allows good map performance at 10–12 levels of zoom.

Both the WHO and JHU CSSE dashboards consider the importance of mobile devices. Among dashboards built with Esri’s ArcGIS Operations Dashboard app, nearly 8% of viewers choose those built for mobile consumption. Consistent with how people want to receive information, mobile-optimised dashboards are versatile and accessible on phones or tablets.

## HealthMap: analysing and mapping online informal sources

**Fig. 3:**
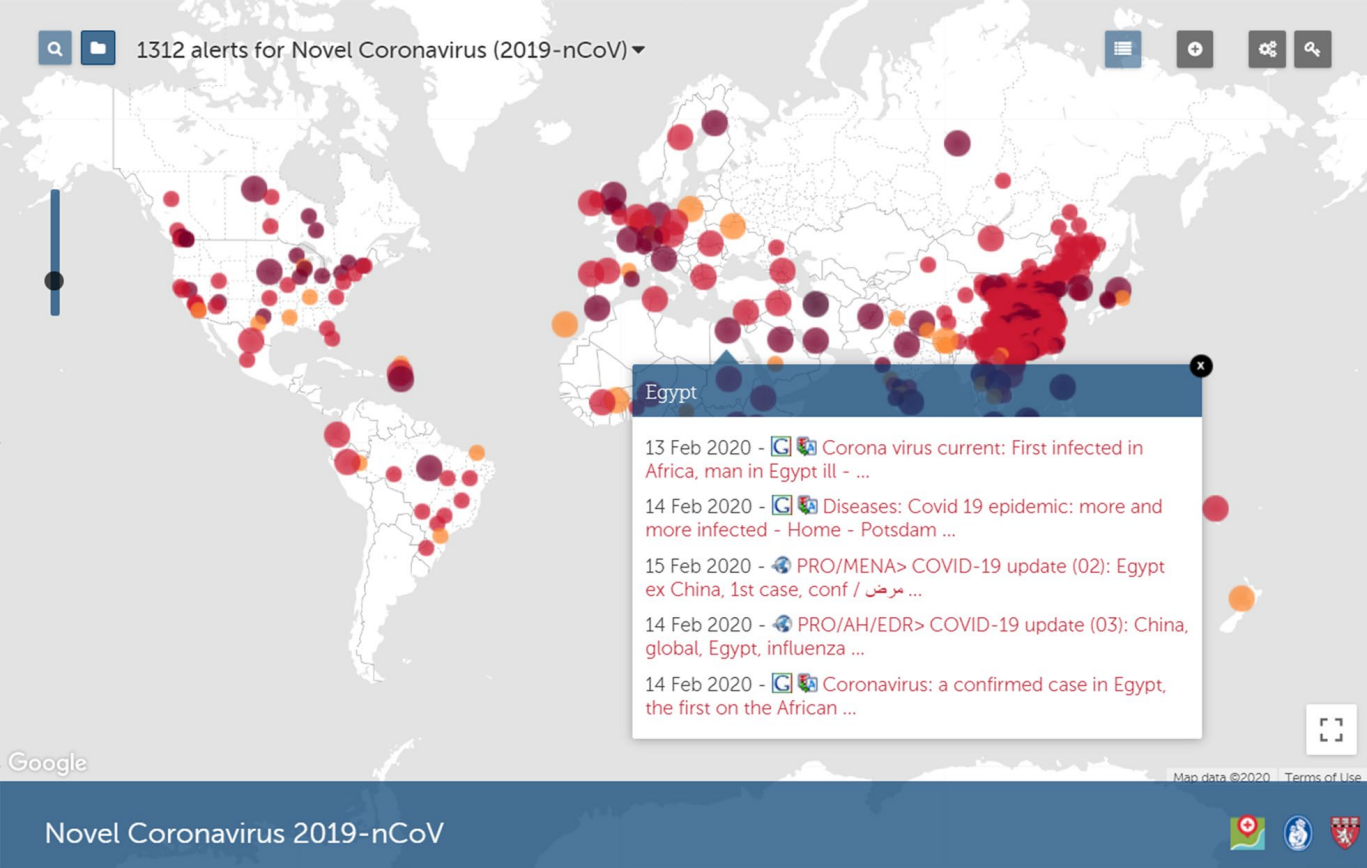
Screenshot of HealthMap for Wuhan Coronavirus showing a number of news articles and alerts about the first case of COVID-19 reported in Africa (Egypt) on 13 February 2020. Screenshot date: 17 February 2020. (HealthMap uses base map data from Google.)

Founded in 2006, HealthMap is run by a team of researchers, epidemiologists and software developers at Boston Children’s Hospital, USA, and uses online media sources for real-time surveillance of emerging public health threats. HealthMap collates outbreak data from a range of sources, including news media (e.g., via Google News), social media, validated official alerts (e.g., from the WHO) and expert-curated accounts [19]. HealthMap’s interactive map for SARS-CoV-2 available at [20] offers near-real-time geolocated updates from these sources to better understand the progression of the pandemic (Fig. 3).

In the same vein as HealthMap, BlueDot, a Canadian firm specialising in automated infectious disease surveillance [21], uses machine learning and natural language processing techniques to sift through news reports in 65 languages, forum and blog posts, airline ticketing data, animal disease networks, etc., to pick up indications and news of unusual, unfolding events and possible disease outbreaks. The firm employs trained epidemiologists to further analyse outbreak results obtained by automated means before releasing them to its clients, and is said to have been among the earliest to break news of COVID-19 outbreak as it started in China.

**Fig. 4:**
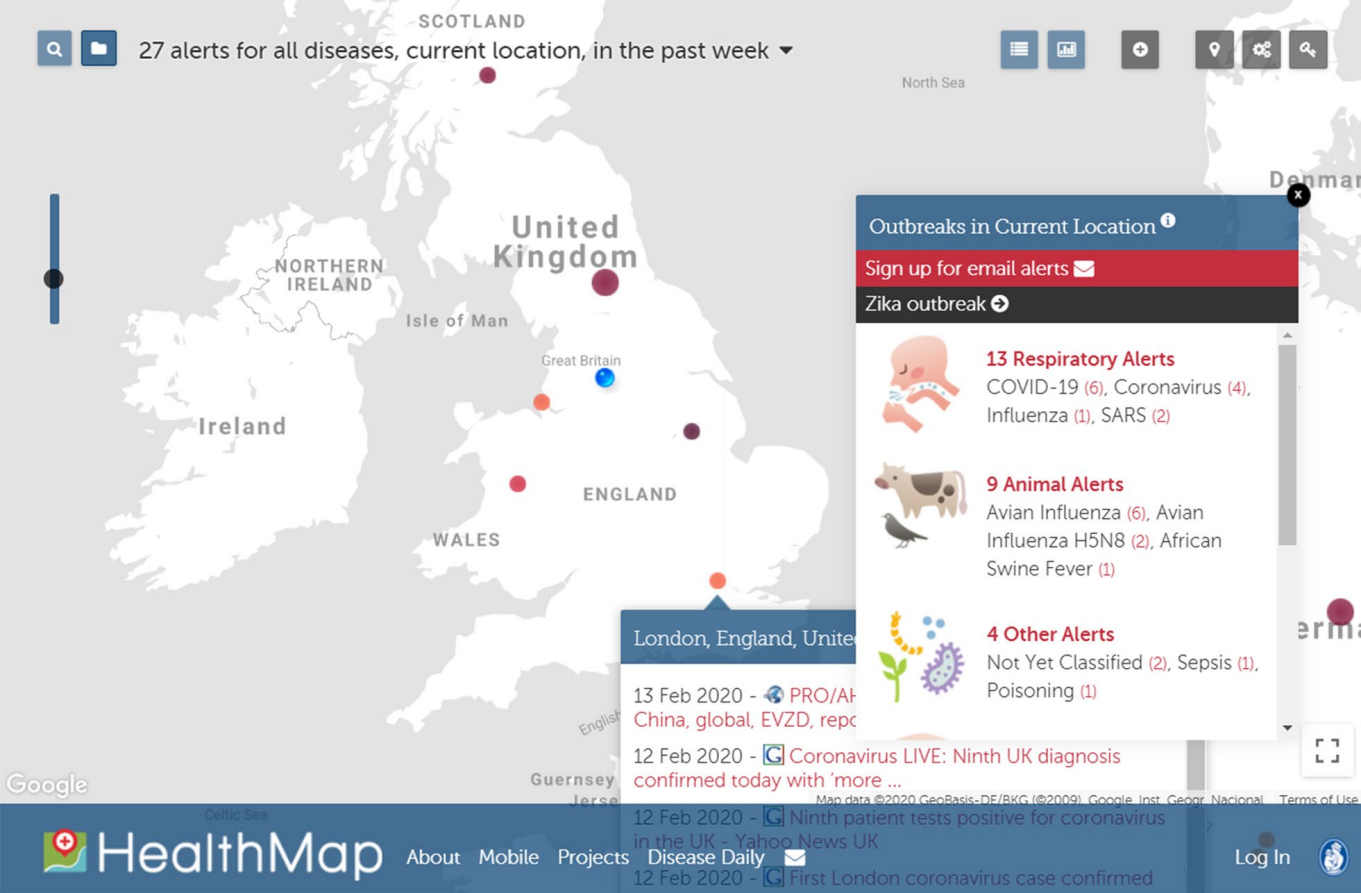
Screenshot of HealthMap’s ‘outbreaks near me’ taken on 17 February 2020. User location has been correctly detected in the United Kingdom, and the expanded news box for London, UK, shows a number of news stories about nearby COVID-19 cases in the UK (nine cases as of 17 February 2020). China’s ‘close contact detector’ platform (see below) expands this concept of ‘outbreaks near me’ in a very much more detailed fashion (much finer location granularity). (HealthMap uses base map data from Google.)

HealthMap also offers an ‘outbreaks near me’ feature that informs individual users about nearby disease transmission risks based on their current location as obtained from their Web browser/smartphone (Fig. 4).

## China’s coronavirus ‘close contact detector’ geosocial app and public service platform

**Fig. 5:**
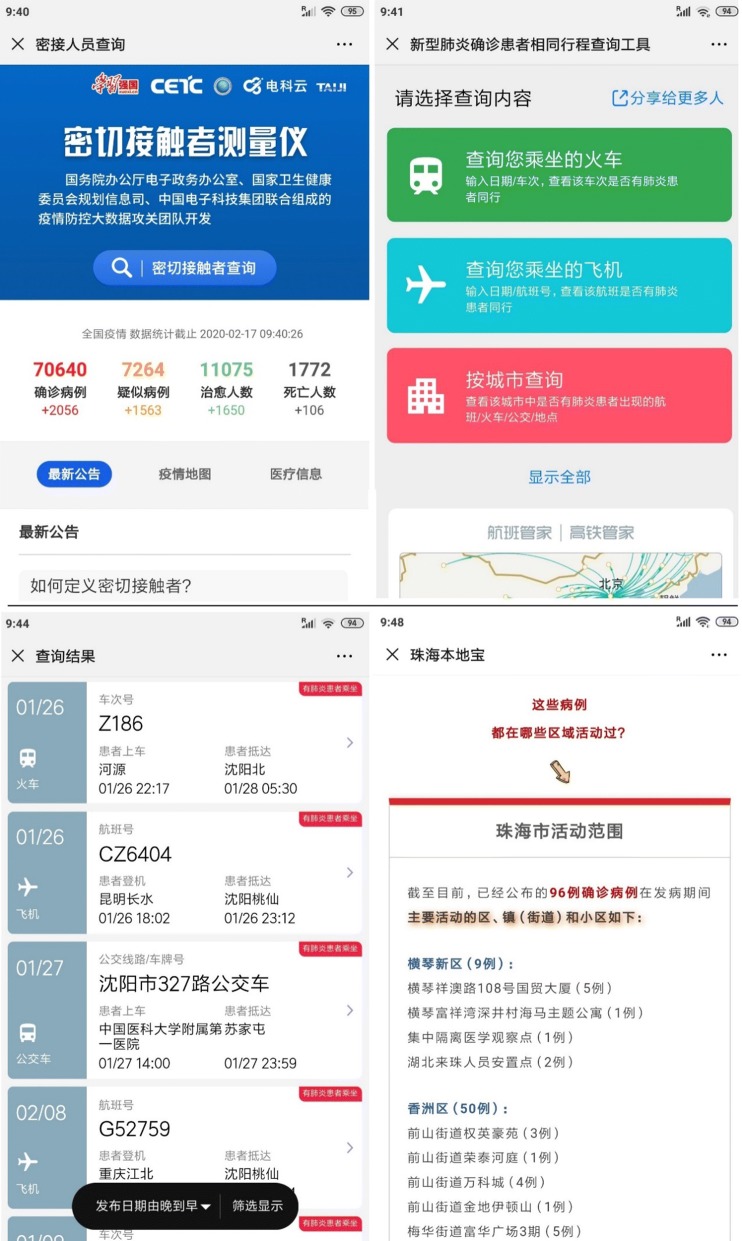
Screenshots of the ‘close contact detector’ app/platform and related online location-based inquiry services in China. Functions include close contact inquiry, including train journey number and plane flight number checking for diagnosed cases, and location information about the activity spaces of nearby confirmed cases (no individual names are ever displayed in returned results). These screenshots were taken on 17 February 2020

While government travel restrictions initiate social distancing, it is now possible for individuals to further the cause by using a dedicated app that provides a detailed spatial scale to support informed personal decisions about self-quarantine and seeking medical treatment. Co-launched by China’s National Health Commission and China Electronics Technology Group Corporation, the ‘close contact detector’ app/platform uses big data from public authorities about the movement of people (public transport data covering flights and trains [booking a train seat in China requires the input of ID information]), as well as disease case records, to check if the user has had any close contact with a person confirmed or suspected to have been infected in the recent past (Fig. 5). The platform can inform the user based on her/his location and recent movements whether s/he has within the last 2 weeks (the assumed incubation period of COVID-19) worked together, shared a classroom, lived in the same building, or travelled by train (all passengers and crew members in the same carriage) or plane (cabin staff and passengers within three rows of an infected person) with a person confirmed or suspected to have the virus. ‘Close contact detector’ can be accessed via three of the most popular mobile social and payment apps in China, namely Alipay, WeChat and QQ [22].

The platform might raise some location data privacy questions among some audiences, even though it explicitly asks its users to observe China’s cybersecurity laws and not abuse private information, and has been very well received by the public in China. As explained by Carolyn Bigg, a Hong Kong-based technology lawyer at the law firm DLA Piper, in a comment to the BBC, “*In China, and across Asia, (individual) data are not seen as something to be locked down, but as something that can be used, provided this is done in a transparent way, with consent where needed. From a Chinese perspective this is a really useful service for people and a really powerful tool that shows the power of data being used for good*” [22].

**Fig. 6:**
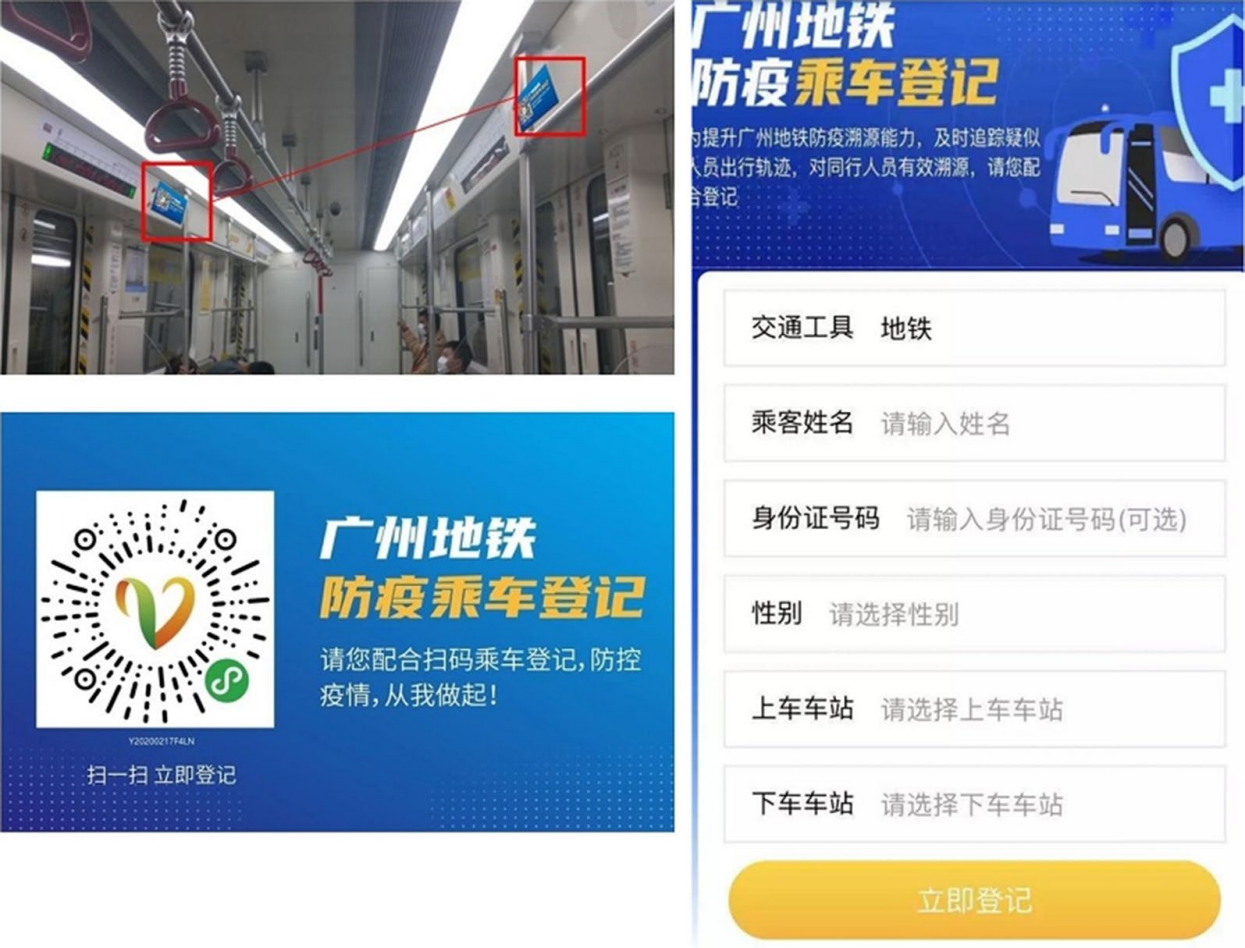
The Guangzhou Underground COVID-19 tracking and notification service. The actual practical value and ultimate success of such services deployed for the first time should be documented and confirmed at the end of the outbreak, so that the world community can learn from the experience

A related and complementary voluntary system was implemented in Guangzhou Underground (Guangdong Province, China), so that if a person is later diagnosed with coronavirus, it would be easier to track her/his transport routines and notify related passengers who boarded the same metro carriages. Starting on 17 February 2020, every metro carriage in Guangzhou Underground displays a unique QR code that passengers are invited to scan once they board the carriage. They then need to quickly fill in an online form that appears on their phone, which includes name, ID No. (optional), gender, their starting metro station and their destination station. As each carriage has a unique QR code, if the passenger walks into another carriage during transportation, s/he will have to scan the new QR code of that carriage and (auto) fill in the corresponding form again. If the passenger’s journey involves changing to other metro lines to reach their final destination, then s/he will have to repeat the same process for every additional carriage they board (Fig. 6).

## WorldPop and EpiRisk predictive global risk analytics and maps for SARS-CoV-2 based on population movements out of Wuhan and travel destinations

Human mobility places scientists at a serious disadvantage in slowing potential epidemics. A person can pick up a virus in one place and share it to another location within hours. Among the jet set, there is potential to become a super spreader [23], infecting many people across an expansive geographical area. While vaccine technology has advanced significantly, it still takes a year or more to formulate a vaccine—time enough for the virus to reach every corner of the world.

**Fig. 7:**
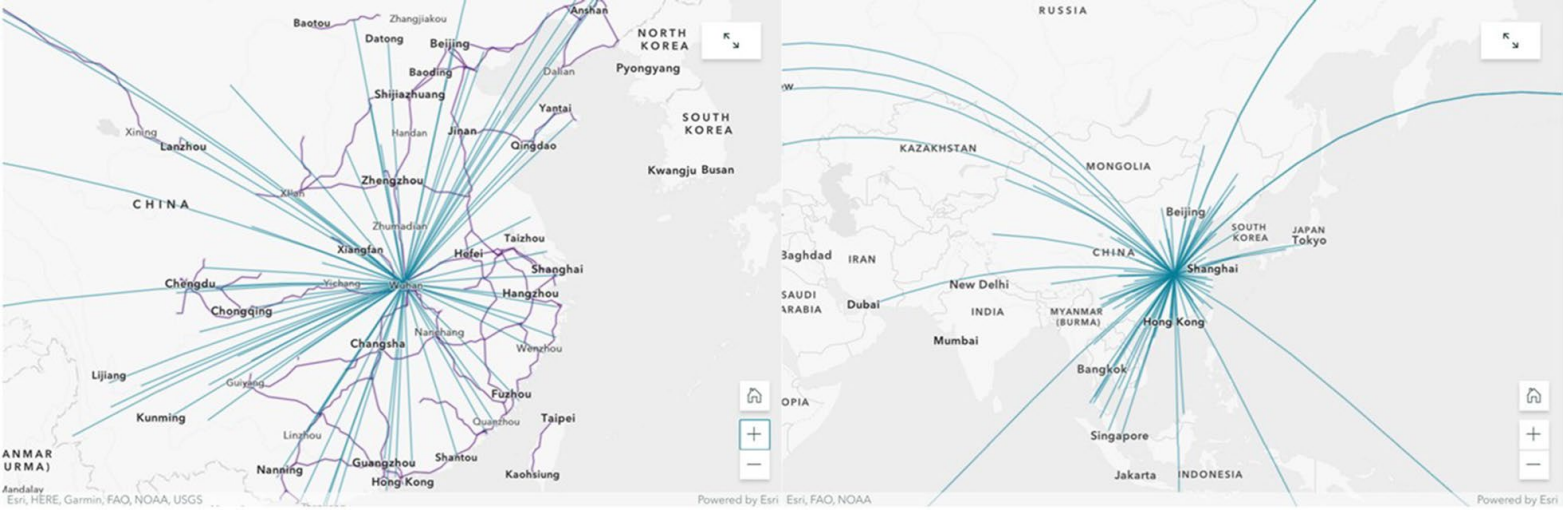
Left: High-speed rail (in purple; 2016) and domestic flights (2018) into and out of Wuhan. Right: International flights leaving Wuhan (partial map; 2018). Wuhan, a major regional transit hub, connects directly to dozens of cities in China. Despite strong actions to curb the spread, an estimated five million people potentially exposed to the virus had already left Wuhan before the city was placed under quarantine, complicating containment efforts. Understanding travel patterns can help health authorities worldwide establish quarantine stations and passenger screening programmes at major international airports

The last trains and scheduled domestic and international flights left Wuhan the morning of 23 January 2020, putting an end to a surge of outbound Chinese Lunar New Year travel that had started 3 days earlier (Fig. 7). The WHO commended the move by Chinese authorities to place Wuhan under quarantine, which was unprecedented at scale. By the end of January 2020, Chinese authorities had enforced further transportation restrictions in 15 additional cities.

The WorldPop research group at Southampton University, UK, used historical data and patterns from Baidu’s location services and international flight itineraries to better understand population movements out of Wuhan prior to the city’s lockdown, look at travellers’ volumes and destinations across China and the world, and compile a predictive global risk map for the likely spread of SARS-CoV-2 virus from its epicentre in Wuhan. According to WorldPop’s analysis, within mainland China, the cities of Beijing, Guangzhou, Shanghai and Chongqing are all identified as high-risk, while the most ‘at-risk’ places worldwide are Thailand (1st), Japan (2nd) and Hong Kong (3rd), followed by the USA (6th), Australia (10th) and the UK (17th). Detailed results and maps can be accessed on the WorldPop online portal [24].

It is noteworthy that WorldPop also mapped population distributions and mobility patterns (mobile phone flow maps using mobile telecom data) in West African countries to support efforts in controlling the 2014 Ebola virus outbreak [25]. Spatial analysis methods are indeed powerful for modelling disease spread, detecting patterns and statistically significant hotspots, and predicting what will happen next [26].

Similar work has been conducted at Northeastern University, Boston, Massachusetts, USA, to develop predictive models of the COVID-19 epidemic using EpiRisk, a tool that estimates the probability that infected individuals will spread the disease to other parts of the world via air travel. Epirisk also tracks the effectiveness of travel bans and is part of the GLEAM (global epidemic and mobility model) project; see their interactive map of COVID-19 at [27].

## Mapping the worldwide spread of misinformation about coronavirus

During infectious disease outbreaks and epidemics, social media play an important role in communicating verified facts and correct prevention tips to the masses, but also carry the risk of ‘virally’ spreading misinformation, confusion and fear among the general public [28, 29]. In the case of COVID-19, false or misleading information, (such as ‘eating sesame oil or garlic can help prevent and cure coronavirus’ and a decade-old map showing global air travel [30]), rumours and panic have been spreading globally on social media much faster than the virus.

**Fig. 8:**
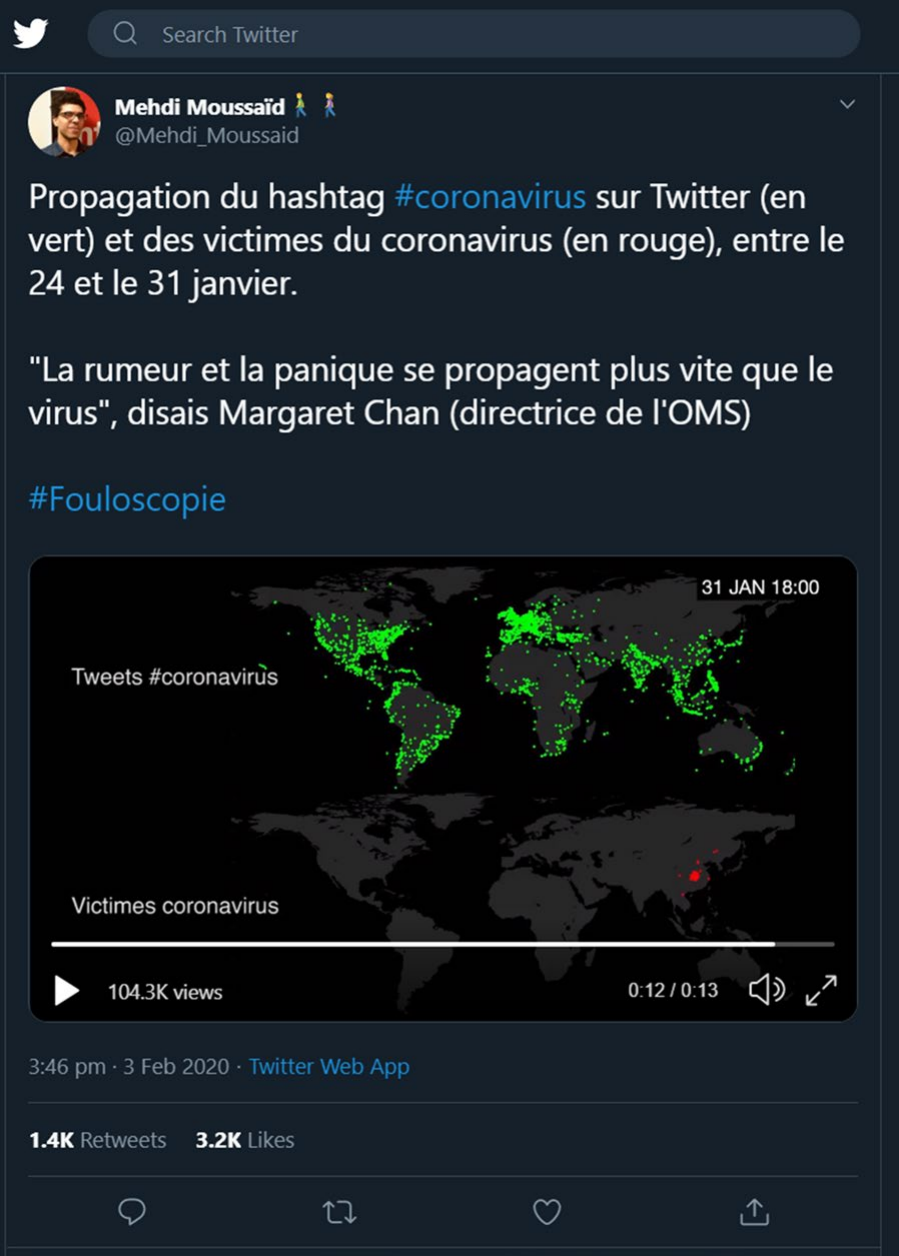
Screenshot of the tweet by @Mehdi_Moussaid on 3 February 2020 featuring an animated map of the worldwide propagation of the hashtag #coronavirus on Twitter (in green) and the actual cases of coronavirus (in red) between 24 and 31 January 2020

To partially illustrate this phenomenon, Twitter user Mehdi Moussaïd (@Mehdi_Moussaid), a research scientist at Max Planck Institute for Human Development, Berlin, Germany, published an animated map of the world on his account showing the worldwide propagation of the hashtag#coronavirus on Twitter (in green) and the actual cases of coronavirus (in red) between 24 and 31 January 2020 (Fig. 8) [31]. Of course, not all tweets and retweets with the hashtag #coronavirus are spreading misinformation, and many of them originate from legitimate bodies and organisations such as the WHO, but the map serves as a good illustration of the ‘viral nature’ of Twitter and other social media. A more detailed map set covering other coronavirus hashtags and classifying tweets by their truthfulness before mapping them could offer valuable insights and guidance for social media companies and health organisations worldwide in their fight against misinformation.

In fact, it has been said that the WHO is fighting a parallel pandemic (or ‘infodemic’) of misinformation besides COVID-19 [32]. The WHO has joined forces with social media giants such as Facebook, Twitter, YouTube (Google) and Pinterest to combat the spread of misinformation around coronavirus. For example, Pinterest and YouTube users can now (at the time of writing this article) see a link prominently displayed that points to an official WHO page about COVID-19 whenever they search for, or browse/watch, material about coronavirus on these platforms.

## Other ways GIS technologies can help in combat infectious disease outbreaks and epidemics

During the COVID-19 outbreak, map-centric dashboards, such as the ones by Johns Hopkins CSSE [12], the WHO [17] and Early Alert Inc. [33], have gone viral themselves, informing both the public and health professionals. But dashboards are just the beginning of how GIS and location technologies can support the fight against infectious diseases. Following are a few more examples.

### Outbreak source

John Snow (1813–1858) was able to trace the source of a cholera outbreak in Soho, London, in 1854, thanks to his well-known manual spatial analysis exercise using hand-drawn paper maps of cholera cases and water pumps/water companies supplying them with water. Today, more advanced computerised spatial analyses integrating phyloepidemiological methods are used to identify the likely sources of new outbreaks; e.g., see the map and discussion of the likely source of SARS-CoV-2 in [34].

### Public events

An important factor affecting epidemics such as COVID-19 is the calendar. During the Ebola and MERS scares of 2014, many people considered cancelling their participation in the Hajj pilgrimage to Mecca made by over two million Muslims every year. Equipped with days-old data and rumours, many faithful proceeded with their pilgrimage, putting themselves at risk of contracting potentially deadly viruses and further spreading disease when they returned home.

In the coronavirus outbreak, Chinese New Year celebrations posed a threat as the themes of togetherness and reunion trigger the largest human migration in the world. The Chinese government extended the Lunar New Year holiday to reduce mass gatherings (that were to happen upon return to work and schools), a public health intervention called social distancing. Travellers worldwide were subsequently restricted from entering China. With access to current information, authorities in Beijing, Macao and Hong Kong cancelled many major festivities.

Dashboards and Web maps that bring together location and time-sensitive events in relationship to a spreading disease give travellers and officials the potential to reduce exposure.

### Site selection

Facing treatment facility shortages in Wuhan, government officials commissioned in late January the emergency construction of two new hospitals, which together provide an additional 2600 beds. Construction teams finished the first hospital on 2 February 2020, just 10 days after breaking ground [35]. The second facility received its first patients on 6 February 2020. Site selection, whether for emergency treatment units or permanent infrastructure, is a common and high-value application of GIS technology.

### Supply chain

During public health emergencies of international concern, we often see shortages of medicines and supplies. Shortages can have significant consequences such as hoarding and price gauging by suppliers or distributors. Sometimes, the impacted places are also manufacturing centres for the supplies, leading to a production decline. Digital supply chain maps prove foundational to planning and ensuring geographical diversity in suppliers as well as aligning needs with distribution.

### Resource locators

Residents of affected areas can use publicly available applications to locate crucial aid and resources. Apps and maps can display information and navigation to hospitals with available beds, clinics offering medical aid along with current wait times, grocery stores and pharmacies that are open, places to purchase personal protective equipment, and more. In heavily impacted cities, this information could critically improve outcomes and save lives.

### Drones

In China, unmanned aerial vehicles (UAV) are transporting crucial medical supplies and patient lab samples. In highly impacted areas, drones reduce human contact with lab samples and free up ground transport assets and personnel [36]. Drones are also being used for broad disinfectant operations in China [37]. Integrated drone and GIS technologies can help target and speed efforts in places they are needed most.

## Conclusions

Modern GIS technologies centre around web-based tools, improved data sharing and real-time information to support critical decision-making. Dashboards exemplify those ideals and have been extremely popular in sharing and understanding the spread of SARS-CoV-2 coronavirus. Communication through map-based dashboards offers accessible information to people around the world eager to protect themselves and their communities. This tool type improves data transparency and helps authorities disseminate information.

Certainly, dashboards have taken centre stage in COVID-19 outbreak awareness. But we hope that readers consider how a comprehensive GIS platform can support the entire process of infectious disease surveillance, preparedness and response, because as one epidemiologist put it, outbreaks like this “*should be expected to happen more frequently moving forward*” [16]. In other words, it is not a question of *if* another outbreak will occur, but *when* and *where*. Viruses like SARS-CoV-2 know no country or continent boundaries.

## Data Availability

Data sharing is not applicable to this article, as no datasets were generated or analysed for the current paper.
